# The effect of action observation on aesthetic preference of Chinese calligraphy: An fMRI study

**DOI:** 10.1002/brb3.2265

**Published:** 2021-06-21

**Authors:** Mingcheng He, Wei Zhang, Jiamin Deng, Xianyou He

**Affiliations:** ^1^ Key Laboratory of Brain Cognition and Education Sciences (South China Normal University), Ministry of Education; ^2^ School of Psychology South China Normal University Guangzhou 510631 China; ^3^ Center for the Studies of Psychological Application South China Normal University Guangzhou 510631 China; ^4^ Guangdong Key Laboratory of Mental Health and Cognitive Science South China Normal University Guangzhou 510631 China

**Keywords:** action observation, aesthetic preference, Chinese calligraphy, fMRI

## Abstract

**Introduction:**

There is some evidence suggesting that movement perception has an effect on aesthetic experience. However, the neural mechanisms underlying the observation of creators’ creative action (the process that calligraphers create calligraphy) remain unclear.

**Methods:**

In this study, participants were scanned with fMRI while performing aesthetic judgments on Chinese calligraphy images with/without action observation.

**Results:**

Behavioral results showed that both the work by the expert and novice with action observation were rated significantly higher on aesthetic preference than those without action observation. Imaging results showed that brain regions associated with perceptual, cognitive, and emotional processing were commonly activated by calligraphy images with/without action observation. However, compared with no action observation, aesthetic judgments of calligraphy images with action observation elicited stronger activation in the anterior cingulate cortex and the bilateral insula. Meanwhile, the superior parietal lobe which is associated with relevant inner action imitation, was also activated when observing the creator's action.

**Conclusions:**

Brain activation in the superior parietal lobe, anterior cingulate cortex, and the bilateral insula indicated that observing the creative action of the creators contributed to the aesthetic experience of the observer.

## Graphical abstract

the neural mechanisms underlying the observation of creators' creative action (e.g., calligraphers creating calligraphy) remain unclear. Our study findings indicate that action observation strongly facilitates observers' aesthetic preferences.

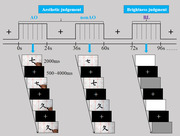

## 1 INTRODUCTION

Action observation refers to the observation of action performed by others (Jeannerod, 2001). Observation of others’ action plays an important role in shaping the perceptual, cognitive, and motor processes of the observer (Welsh et al., 2009). Many studies have found that action observation could improve motor skill learning (Gonzalez‐Rosa et al., 2014; Rohbanfard & Proteau, 2011). For example, without physical practice, observers improved their dance skills through action observation (Cross et al., 2009), because action observation provided a “perceptual blueprint” (Cross et al., 2009; Sheffield, 1961). In addition, the predictive coding model provided another possible explanation. Kilner et al. (2007) proposed that, given an expectation of the goal of the person being observed, an observer could predict the motor commands and further predict the kinematics based their own action system. The comparison of the predicted kinematics with the observed kinematic generated an error. Then, the inferred goals were updated by minimizing the error between the predicted and the inferred motor commands, until the most likely cause (e.g., intention and goal) of the observed action was inferred. According to the predictive coding model, when watching others in action (e.g., dance), we would make a prediction for the action and readily infer their intentions from subtle changes in the way they move, and eventually affect the observer's own behavior. For example, observing others’ gestures influenced the observers’ reasoning (Alibali et al., 2014 ), because they could effectively perceive their surroundings from the others’ perspective (Frischen et al., 2009 ). In other words, action observation could help observers comprehend the action, enhance empathy, and further affect their affective response (Cole et al., 2015; Frischen et al., 2009; Molnar‐Szakacs, 2011; Spunt et al., 2011), which were closely related to aesthetic experience (Freedberg & Gallese, 2007; Kirsch et al., 2016 ). Also, Kirsch et al. (2016) found that perceiving others in action elicited affection of observers. Further, based on the previous literature, we would like to know whether such benefits would transfer to aesthetic experience, and assume that there is a possibility that observers benefit not only in promoting action performance but also in aesthetic experience by action observation.

In the visual arts, previous studies have mainly examined the influence of the movement characteristics of aesthetic objects on observers’ aesthetic preferences. One approach incorporated real movement in the object of appreciation. For example, Zhao et al. (2020) found that the participants preferred the real moving landscapes to the static landscapes. The other approach involved implied action in the content of the object of appreciation. For example, Di Dio et al. (2016) found that both dynamic characteristics and landscapes had stronger activation in motor‐related brain regions. These studies indicated that the movement characteristics implied in the object of appreciation could affect participants’ aesthetic experiences. However, few studies have paid attention to movement characteristics outside the object of appreciation, like the creative action of the creator. Creative action is the action when the creator is creating the artwork. In fact, some researchers have proposed that perceiving the creative action of creators may be closely related to observers’ emotional responses (Freedberg & Gallese, 2007; Ticini et al., 2015; Tinio, 2013). Freedberg and Gallese (2007) suggested that observers would spontaneously imagined the action of artists from the creative traces of visual art, such as knife marks left on marble statues and brushstrokes on the canvas. The neuroimaging study by Di Dio et al. (2011) found that motor‐related brain regions (e.g., premotor cortex) were significantly activated when participants appreciated marble statues. Taylor et al. (2012) provided evidence showing that observers could simulate the actions of the painter by simply observing brushstrokes. Furthermore, Ticini et al. (2014) adopted visual training in which pictures were presented with different pen‐holding gestures to prime an action. The results showed that when the gesture pictures were congruent with the artist's painting style, participants tended to have higher aesthetic preference ratings. Williams et al. (2020) trained participants to associate painting actions with hand primes to enhance visuomotor and visuovisual associations of artist actions. The results showed that compared with the incongruent priming condition, actions congruent with the artist increased observers’ fixation time. These previous behavioral studies suggest that perceiving creative action might positively impact observers’ aesthetic experience.

In addition, prior studies suggested that observing others’ action induced observers’ inner imitation desires (Lebreton et al., 2012) or facilitated observers’ covert simulation of the creators’ action (Ticini et al., 2017), which could help observers infer the cause of action (Kilner et al., 2007 ), comprehend the action, enhance empathy, and further affect their affective response (Cole et al., 2015; Freedberg & Gallese, 2007; Frischen et al., 2009; Kirsch et al., 2016; Molnar‐Szakacs, 2011; Spunt et al., 2011). Moreover, fluency theory (Reber et al., 2004) proposes that positive aesthetic experiences are linked to processing ease. Therefore, compared with the indirect perception elicited by cues of brushstrokes (Freedberg & Gallese, 2007), direct observation of the creator's action was easier to process. Thus, we hypothesize that observing creative action performed by creators would elicit observers’ inner action imitation and further promote their emotional response to the object of appreciation. The activities in the brain should be reflected in stronger activation of brain regions associated with action observation and aesthetic experience.

We use Chinese calligraphy images as aesthetic materials in this study for the following reasons. First, Chinese calligraphy has high aesthetic value and is one of the symbols of Chinese culture; however, the behavioral and neural manifestations of the aesthetic experience associated with Chinese calligraphy remain unclear. Second, Chinese calligraphy is an art form that emphasizes the creative action of writer (Xu et al., 2016). Novices in Chinese calligraphy need to observe the instructor's creative action to improve their own writing skill, which includes the precise writing of each stroke, and the rhythm of writing (Chen et al., 2017). Finally, the appreciation of creators’ on‐site creation is a common form of calligraphy appreciation in China. Furthermore, Lebreton et al. (2012) found that observers had stronger desire to imitate the observed action when action‐directed goal was attractive (e.g., toy). However, we do not know whether observers would reduce their inner imitation desire when the action‐directed goal is unattractive. Thus, we would use the creator's work with different artistic levels, i.e., calligraphy expert’ and novice’. In one word, Chinese calligraphy is a suitable artistic medium to examine the influence of action observation on observers’ aesthetic preferences.

In the present study, we attempted to investigate the neural substrates of the appreciation of Chinese calligraphy in different action observation conditions. Specifically, if our inferences are reasonable, we can predict the significant activation in motor‐related regions such as the parietal lobe while viewing creators’ creative actions. For example, several studies have reported that the premotor and the superior parietal lobule were significantly activated during action observation (see meta‐analysis by Hardwick et al., 2018). Meanwhile, prior studies suggested that multiple functions were involved in aesthetic judgment, including perceptual processing, cognitive judgment, aesthetic emotional, and rewarding processing (see meta‐analysis by Boccia et al., 2016). Many neural studies have associated perceptual processing of beauty with activity in the occipital lobe such as the middle occipital gyrus and fusiform gyrus, regions that involve visual preference and attention modulation. Cognitive judgments of beauty were associated with activation in the frontal lobe such as the middle and inferior frontal gyrus. Emotional and rewarding processing are major components for aesthetic judgments, and guide motivation and decision‐making (Vartanian et al., 2013; Zhang et al., 2019). For example, the imaging results in Zhang et al. (2017) revealed regions associated with perceptual, cognitive, emotional, and rewarding processing were commonly activated both in beautiful judgments of pictographs and oracle bone scripts.

According to the above assumption, if the processing of action observation indeed contribute to observer's aesthetic experience, we could observe not only the activation of brain regions associated with perception and judgment processing, but also the significant activation of emotional and rewarding processing regions, like anterior cingulate cortex (ACC), insula, etc. (Boccia et al., 2016; Di Dio et al., 2016; Skov & Nadal, 2020) which relate to aesthetic judgments. Previous studies (Brielmann et al., 2017; Verhavert et al., 2018) also indicated that observers perceived beauty very fast and almost automatic. For example, Verhavert et al. (2018) found that aesthetic judgment could be formed based on very glances of information (about 50 ms). Meanwhile, concerning existing research designs on the aesthetics of Chinese characters (see Zhang et al., 2016, 2017), this study aims to explore the relationship between action observation and aesthetic judgment within 2 s.

## 2 METHOD

### 2.1 Participants

We recruited 27 healthy right‐handed college students (10 males, 17 females) between 19 and 24 years of age (*M*_age _= 21.7, *SD *= 2.43) for this experiment. All participants had normal or corrected‐to‐normal vision. None of them had received professional training in calligraphy. Written informed consent was obtained from each participant, and the protocol was approved by the Institute Ethics Committee, South China Normal University.

### 2.2 Experimental procedures

#### 2.2.1 Stimuli

**FIGURE 1:**
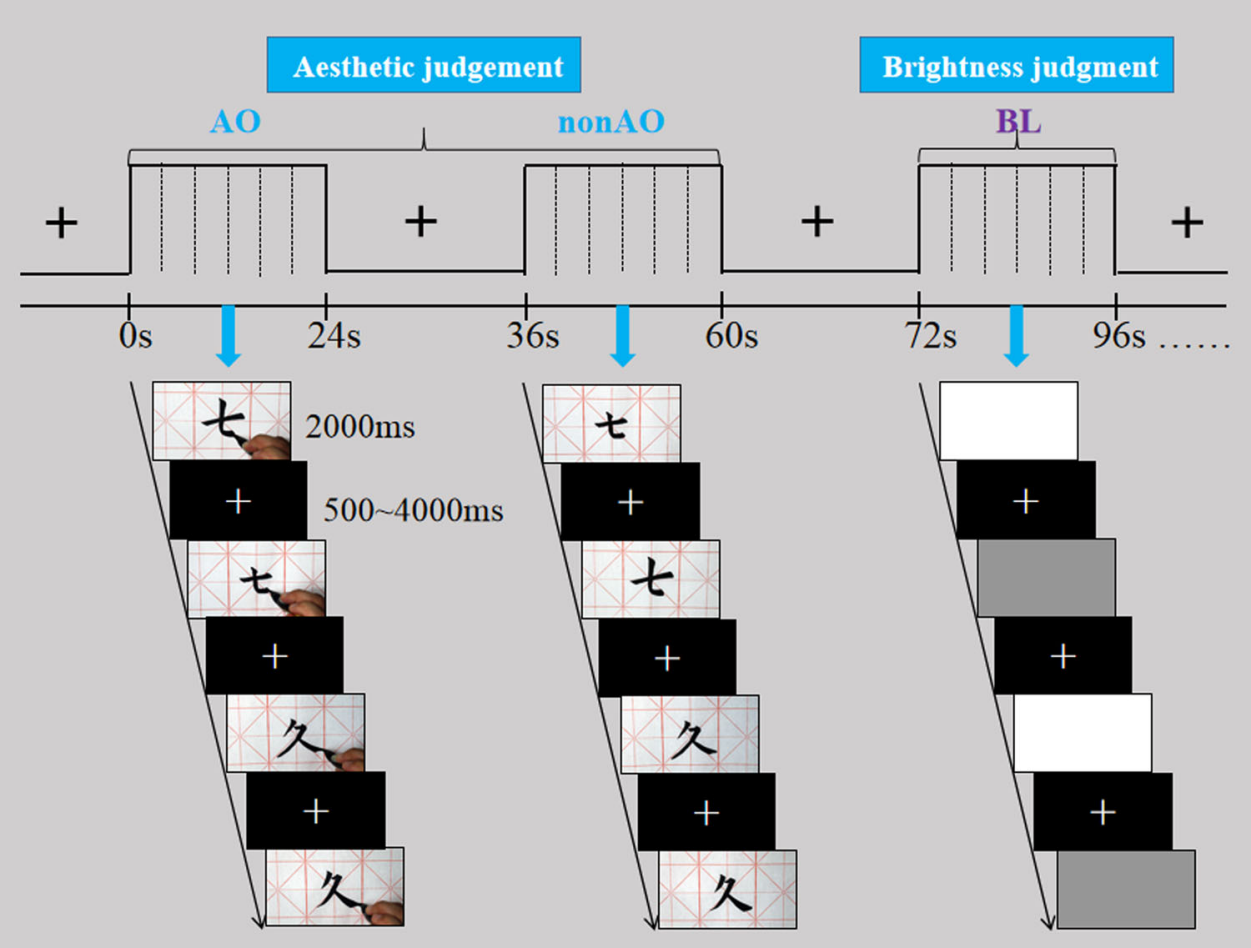
Experimental design, procedure, and examples of stimuli. Two types of tasks were performed in separate blocks: aesthetic judgments (expert/novice work with/without AO) and baseline judgments (high/low luminance)

We created three stimuli categories: calligraphy images with/without action observation, calligraphy images by one expert/novice, and high/low luminance gray squares. The stimuli consisted of 96 images of brush‐pen calligraphy and 48 gray squares. First, a calligraphy expert and a calligraphy novice were invited to separately write 24 characters, where the number of strokes ranged from two to four (*M *= 3.63, *SD *= 0.59). Then, a total of 48 brush‐pen calligraphy image pairs were selected as the stimulus set. Another 26 participants (6 males, 20 females, *M*_age _= 20.46, *SD *= 1.86) used a seven‐point scale (1 means the calligraphy work is very ugly, and 7 means the work is very beautiful) to rate the beauty of 48 Chinese characters. Results showed that the average beauty rating for the experts’ work (*M *= 6.50, *SD *= 0.83) was significantly higher than the novices’ work (*M *= 3.64, *SD *= 0.88), *t*(58) = 12.95, *P *< 0.001. For each pair, two images were photographed. One image included the writing hand to create the condition of action observation, while the other image did not include the writing hand to create the condition of not observing the action (refer to Lebreton et al., 2012). Examples of materials are shown in Figure 1. The reasons for this treatment are as follows: On the one hand, we believe that this treatment not only allows observers to watch the creator's action but also ensures the integrity of the work, which allows the action‐directed goals in the with/without action observation condition to remain almost uniformly similar. On the other hand, previous views or studies have also suggested that observing action in static pictures could also induce action simulation by the observer (Freedberg & Gallese, 2007; Di Dio et al., 2016; Kolesar et al., 2016; Ticini et al., 2017). So we believe that this treatment shows that the work is in a state of completion but not yet finished, and those Chinese participants who know the rules of writing Chinese characters would have the desire to continue to complete the work, thus creating a motor plan. This creates a realistic feeling as if one was observing the creator's live creation. Thus, four photographs were made for each pair of objects: two artistic levels (expert/novice work) × two observation conditions (with/without action observation). Finally, to control the valence of the characters, the same 26 participants used a seven‐point scale (1 means the meaning of Chinese characters is very negative, and 7 means the meaning of Chinese characters is very positive) to rate the valence of the Chinese characters between the four conditions. Results showed that there was no statistical difference of the average valence between the four conditions *F*(3, 100) = 0.66, *P *= 0.57. For gray squares, we used 24 high luminance gray squares (RGB = 255, 255, 255) and 24 low luminance gray squares (RGB = 64, 64, 64), which were generated according to standard RGB values by using Adobe Photoshop 7.0 software (Zhang et al., 2016). All images were featured in high resolution (534 × 300 pixels) for the rating task, and the stimuli were presented with E‐prime 2.0 (Schneider et al., 2002) and displayed on an LCD projector (resolution: 1920 × 1080 pixel, 60 Hz refresh rate) at a distance about 100 cm to the participant's eyes, covering a visual angle of approximately 4° vertical × 8° horizontal. Samples of the materials and the experimental procedures for the present study are illustrated in Figure 1.

#### 2.2.2 Task

During the scanning, each participant was asked to perform aesthetic judgments and square luminance judgments. The participants were instructed to judge whether they would like the calligraphy images with/without action observation. The aesthetic judgment condition required the participants to press one of two buttons to indicate whether they like the stimulus. The square luminance judgment condition served as a baseline, and the participants were instructed to judge whether the luminance of the square was high or low by pressing one of two buttons. The finger‐response mapping was counterbalanced across the participants.

#### 2.2.3 Procedure

The scanning session included experimental conditions (expert/novice work with/without action observation) and baseline conditions (low/high luminance). Each condition included 48 trials and was presented with one repetition. We used a hybrid design with 16 blocks for each condition. Block orders were fixed and counterbalanced across participants. Each block lasted for 24 s and was followed by a 12‐s fixation interval. Each block contained six trials. Within each block, the presentation of each trial lasted 2 s including response time in pseudo‐random order (event‐related design), with a variable jitter times of 500–4000 ms as the interstimulus interval (ISI).

#### 2.2.4 Data acquisition

MRI data were acquired on a 3T Siemens Prisman Fit scanner with a 20‐channel phased‐array head coil at the Magnetic Resonance Imaging Lab, South China Normal University. A gradient echo‐planar imaging sequence was used with the following parameters: slice thickness = 3 mm, interslice gap = 1 mm, TE = 30 ms, TR = 2000 ms, flip angle = 90°, matrix size = 64 × 64, FOV = 192 mm, and 32 axial slices covering the whole brain. T1‐weighted 3D structural images were acquired by using an MP‐RAGE sequence: TE = 2.52 ms, TR = 2530 ms, flip angle = 7°, voxel size = 1 × 1 × 1 mm^3^.

#### 2.2.5 Data analysis

Data preprocessing and analysis were performed using SPM8 (http://www.fifil.ion.ucl.ac.uk/spm/). For stabilization of magnetization, the first five volumes of each session were discarded. The remaining images were corrected for slice timing and spatially realigned to the first volume for correcting head movements. One participant was excluded from the subsequent analysis, as the individual's images had > 2 mm maximum displacement and > 1.5° rotation. The other preprocessing steps were as follows: the T1‐weighted image were coregistered to the mean echo‐planar images, a mean image created from realigned volumes was spatially normalized to the MNI echo‐planar imaging brain template using nonlinear basis functions, and resampling with a voxel size of 3 × 3 × 3 mm^3^. The normalized functional images were smoothed with an isotropic 6‐mm full width‐half‐maximum (FWHM) Gaussian kernel.

In the first‐level analysis, a general linear model was applied to the fMRI time series in which stimulus onset was modeled as a single impulse response function and then convolved with the canonical hemodynamic response function. We modeled seven regressors of interest: calligraphy work with action observation (AO), calligraphy work without action observation (nonAO), expert work with action observation (AO_EW), novice work with action observation (AO_NW), expert work without action observation (nonAO_EW), novice work without action observation (nonAO_NW), and square luminance (SL). Head movement parameters calculated from the realignment procedure were included in the model as covariates of no interest. A high‐pass filter with a cutoff of 128 s was applied to remove low‐frequency signal drifts.

Contrast images for aesthetic judgments of calligraphy images and luminance judgments of gray squares were taken to second‐level *t* tests and modeled into flexible factorial analyses. The main interest in this present study was to identify the cortical networks involved in the aesthetic judgments of calligraphy images with/without AO. We therefore utilized the SL judgment as a baseline to control for activity in motor brain regions associated with the key responses. We first performed the contrasts of “nonAO > SL,” “nonAO_EW > SL,” “nonAO_NW > SL,” “AO > SL,” “AO_EW > SL,” and “AO_NW > SL.” Moreover, direct comparisons of “AO > nonAO,” “AO_EW > nonAO_EW,” and “AO_NW > nonAO_NW” were conducted to investigate differences in neural mechanisms between aesthetic judgments of calligraphy images with/without AO. For the above models, global analyses were conducted with a voxel threshold of *P *< 0.001 (uncorrected) and a cluster threshold with FDR < 0.05.

## 3 RESULTS

### 3.1 Behavioral results

#### 3.1.1 Reaction rates for aesthetic judgments

The Shapiro–Wilk test (Shapiro & Wilk, 1972 ) shows that data were not normally distributed (*P *< 0.05). Therefore, we did the log‐transformed before statistical analysis (Csibra et al., 2016). Then, a 2 (observation condition: with/without AO) × 2 (artistic level: expert/novice work) repeated‐measures ANOVA with the rates of liking rating as the dependent variable revealed a significant main effect of observation condition, *F*(1, 25) = 8.42, *P =* 0.008, *η_p_* ^2^ *^^*= 0.25, and a significant main effect of artistic level, *F*(1, 25) = 134.55, *P *< 0.001, *η_p_* ^2^ *^^*= 0.84. Meanwhile, we also found a significant interaction between observation condition and artistic level, *F*(1, 25) = 5.48, *P =* 0.028, *η_p_* ^2^ *^^*= 0.18.

A simple effect test (Bonferroni post hoc test) showed that for the expert work, the participants had a higher “liking” response in the condition with AO (0.82 ± 0.02) than in the condition without AO (0.79 ± 0.02), *F*(1, 25) = 6.93, *P *= 0.014, *η_p_* ^2 ^= 0.22. However, for the novice work, the participants had marginally higher “liking” responses in the condition with AO (0.28 ± 0.04) than in the condition without AO (0.18 ± 0.03), *F*(1, 25) = 3.77, *P =* 0.064, *η_p_* ^2^ *^^*= 0.13. These results suggested that AO contributed to the observers’ aesthetic preferences, especially when the participants observed the creative action of the expert.

### 3.2 fMRI results

#### 3.2.1 Calligraphy images without AO versus baseline

**FIGURE 2:**
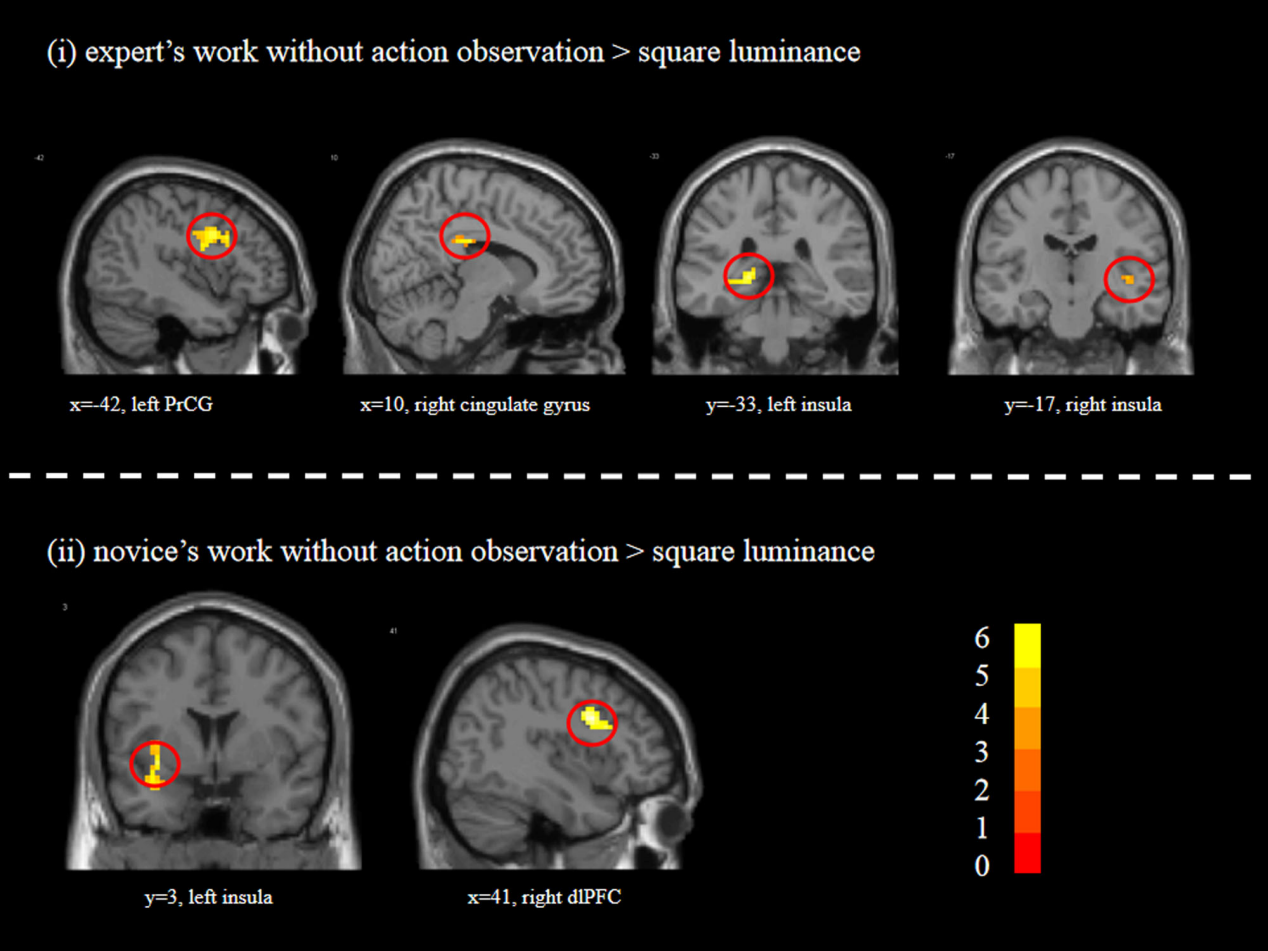
The main cerebral areas for aesthetic judgments of calligraphy images without AO. *t* scores are rendered in colors ranging from 0 (red) to positive (yellow) as indicated by the accompanying color bar

In contrast of “nonAO > SL,” we found that aesthetic judgment of calligraphy images without AO was associated with activity in the middle occipital gyrus, inferior occipital gyrus, cingulate gyrus, and bilateral insula. The contrast “nonAO_EW > SL” resulted in activities in the inferior occipital gyrus, inferior frontal gyrus, precentral gyrus, cingulate gyrus, and bilateral insula. For the contrast of “nonAO_NW > SL,” we observed stronger activations in the middle occipital gyrus, inferior occipital gyrus, middle frontal gyrus, dorsolateral prefrontal cortex, and insula (see Table 1 and Figure 2).

**TABLE 1:** Brain regions of the analysis of variance between aesthetic judgments of calligraphy images without AO (*P* < 0.05, FDR corrected)

		Peak MNI coordinates				
Brain regions	Hemisphere	*x*	*y*	*z*	*t* score	Cluster size
**NonAO > SL**						
MOG	R	42	−78	−3	13.81	438
IOG	L	−33	−87	−9	14.74	110
Cingulate gyrus	R	9	−12	27	4.58	47
Insula	L	−33	18	3	4.35	41
	R	39	−3	9	5.38	47
**NonAO_EW > SL**						
IOG	L	−36	−87	−12	12.42	104
IFG	R	48	9	27	5.62	351
PrCG	L	−51	−9	33	4.82	120
Cingulate gyrus	R	15	−30	27	5.78	33
Insula	L	−33	15	3	4.41	30
	R	39	−3	6	4.20	20
**NonAO_NW > SL**						
MOG	R	42	−78	−3	11.56	377
IOG	L	−33	−87	−9	13.56	103
MFG	R	27	−6	48	3.68	41
dlPFC	R	45	9	30	5.78	57
Insula	L	−30	18	3	4.98	68

Abbreviations: dlPFC, dorsolateral prefrontal cortex; IFG, inferior frontal gyrus; IOG, inferior occipital gyrus; MFG, middle frontal gyrus; MOG, middle occipital gyrus; PrCG, precentral gyrus.

#### 3.2.2 Calligraphy images with AO versus baseline

**FIGURE 3:**
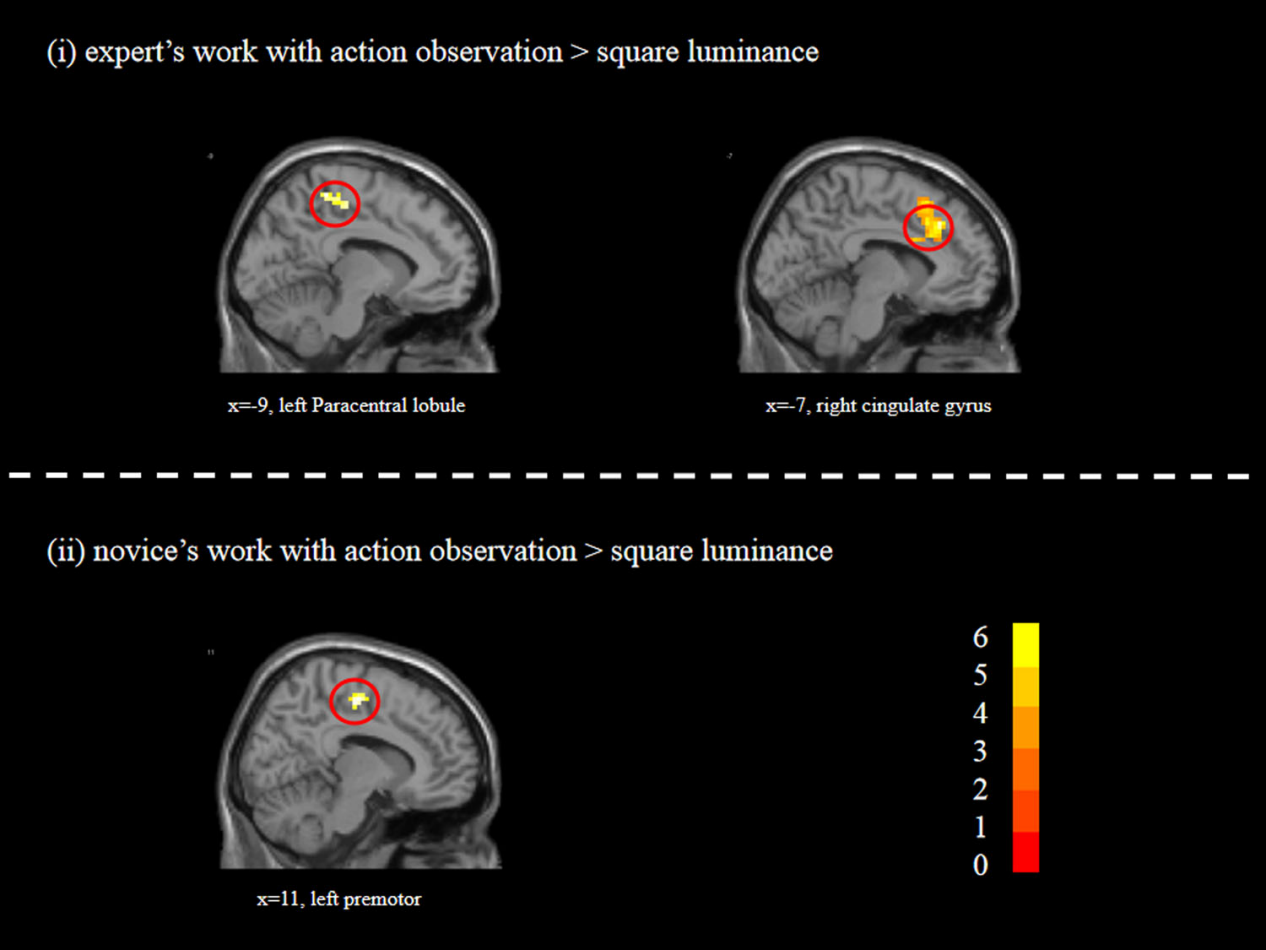
The main cerebral areas for aesthetic judgments of calligraphy images with AO. *t* scores are rendered in colors ranging from 0 (red) to positive (yellow) as indicated by the accompanying color bar

In contrast of “AO > SL,” we found that aesthetic judgment of calligraphy images with action observation was associated with activity in the fusiform gyrus, dorsolateral prefrontal cortex, premotor, and cingulate gyrus. In the contrast “AO_EW > SL,” we found that judgments of expert work were associated with activities in the inferior occipital gyrus, middle frontal gyrus, paracentral lobule, and cingulate gyrus. For the contrast of “AO_NW > SL,” we observed stronger activations in the inferior occipital gyrus and premotor (see Table 2 and Figure 3).

**TABLE 2:** Brain regions of the analysis of variance between aesthetic judgments of calligraphy images with AO (*P* < 0.05, FDR corrected)

		Peak MNI coordinates				
Brain regions	Hemisphere	*x*	*y*	*z*	*t* score	Cluster size
**AO > SL**						
Fusiform gyrus	L	−45	−69	−3	15.8	181
	R	42	−51	−18	14.0	202
dlPFC	R	57	15	30	7.97	87
Premotor	L	30	−6	54	4.78	87
Cingulate gyrus	R	12	24	39	6.44	97
**AO_EW > SL**						
IOG	L	−30	−81	−9	10.96	198
MFG	R	27	−3	63	4.19	107
Paracentral lobule	L	−9	−39	60	3.30	21
Cingulate gyrus	R	9	24	39	5.04	95
**AO_NW > SL**						
IOG	L	−30	−84	−12	9.56	201
Premotor	L	12	−15	54	3.73	7/28

Abbreviations: PrCG, precentral gyrus; SMA, supplementary motor area.

#### 3.2.3 Calligraphy images with versus without AO

**FIGURE 4:**
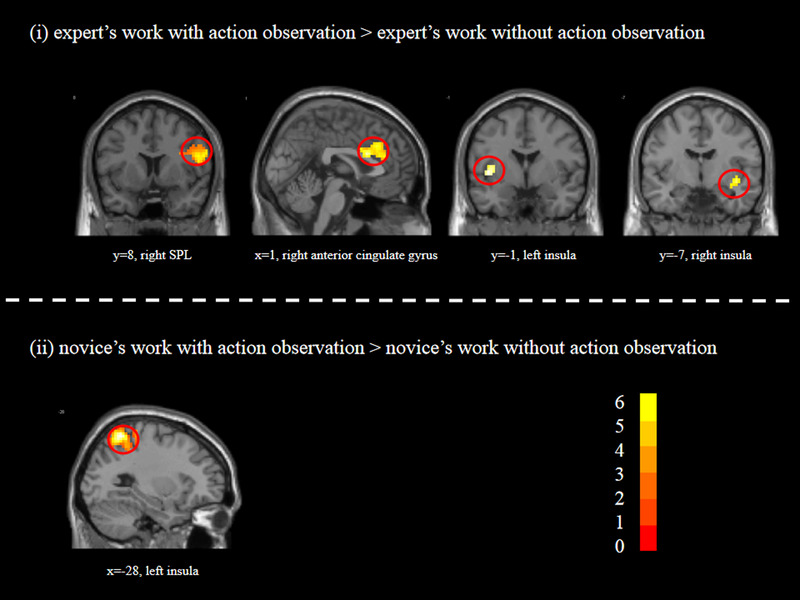
The main cerebral areas for aesthetic judgments of calligraphy images with vs. without AO. *t* scores are rendered in colors ranging from 0 (red) to positive (yellow) as indicated by the accompanying color bar

To investigate whether the neural mechanisms of the aesthetic judgment in the condition with AO were different from those in the condition without AO, a direct comparison was conducted between the aesthetic judgments in the conditions with AO and without AO. Significant differences were found in the contrasts of “AO > nonAO,” “AO_EW > nonAO_EW,” and “AO_NW > nonAO_NW.” In contrast of “AO > nonAO,” we found that aesthetic judgment of calligraphy images with action observation was associated with activity in the fusiform gyrus, middle occipital gyrus, and superior parietal lobule. In the contrast “AO_EW > nonAO_EW,” we found that aesthetic judgments of the expert's work with action observation were associated with activations in the middle occipital gyrus, superior parietal lobule, inferior frontal gyrus, anterior cingulate gyrus, and bilateral insula. For the contrast of “AO_NW > nonAO_NW,” we observed stronger activations in the fusiform gyrus, superior parietal lobule, middle temporal gyrus, and postcentral gyrus (see Table 3 and Figure 4).

**TABLE 3:** Brain regions of the analysis of variance between aesthetic judgments of calligraphy images with and without AO (*P* < 0.05, FDR corrected)

		Peak MNI coordinates				
Brain regions	Hemisphere	*x*	*y*	*z*	*t* score	Cluster size
**AO > nonAO**						
MOG	L	−24	−87	3	11.17	485
Fusiform gyrus	R	51	−66	0	12.90	88
SPL	L	−30	−60	57	9.60	151
**AO_EW > nonAO_EW**						
MOG	L	−27	−90	3	7.02	442
SPL	R	30	−60	60	6.43	125
IFG	L	−45	9	24	3.13	32
ACC	R	6	15	27	4.90	75
Insula	L	−42	0	3	3.57	44
	R	33	15	6	2.88	46
**AO_NW > nonAO_NW**						
Fusiform gyrus	L	−48	−69	0	9.76	48
SPL	L	−27	−54	60	11.09	146
MTG	R	51	−57	0	7.11	187
PoCG	R	48	−24	36	5.96	68

Abbreviations: MTG, middle temporal gyrus; PoCG, postcentral gyrus; SPL, superior parietal lobule.

## 4 DISCUSSION

The present study used Chinese calligraphy as materials to investigate the effect of observing the creator's creative action on the participants’ aesthetic judgment. The behavioral results revealed that calligraphy images with AO were more likely to be judged as “like” than those without AO, especially in the condition of the expert's work with AO.

Consistent with previous studies, in our neuroimaging results, we found that the middle occipital gyrus (MOG) and inferior occipital gyrus (IOG) were activated for aesthetic judgments of Chinese images with/without AO. The occipital cortex is often considered a neural substrate underlying the modulation of sensory processing (Chatterjee & Vartanian, 2016), such as visual attention to objects (Nadal, 2013), shape and color (Boccia et al., 2016; Vartanian & Skov, 2014; Zhang et al., 2016). In fact, the occipital cortex also reflected the observers’ aesthetic preference. For example, Vartanian and Skov (2014) proposed that structures involved in visual perception could contribute to the computation of preferences. Specially, we observed fusiform gyrus activation in contrast of “AO > SL,” “AO > nonAO,” and “AO_NW > nonAO_NW,” which could be attributed to the processing of the agent's hand under the condition of action observation, for previous studies have shown that the fusiform gyrus encodes images of the human body (Peelen & Downing, 2004; Schwarzlose et al., 2005).

In addition, we observed that the middle frontal gyrus (MFG) was activated in contrast of “nonAO_NW > SL” and “AO_EW > SL.” The activation of inferior frontal gyrus (IFG) was found in the contrast of “AO_EW > nonAO_EW” and “nonAO_EW > SL,” respectively. Previous imaging results revealed that the frontal cortex was primarily involved in cognitive judgment and emotional processing (Boccia et al., 2016; Zhang et al., 2016). Zhang et al. (2016) have reported that the bilateral IFG were activated for aesthetic judgments of beauty for both the pictographs and the object images. Yeh et al. (2015) also found that subjective ugliness and negative emotion both activated the right IFG. Furthermore, the activation of MFG, IFG, and middle temporal gyrus (MTG) may also be involved in language processing. For example, large parts of the frontal lobe and MTG were activated when participants were requested to read several proverbs (Bohrn et al., 2013), poetry (Gao & Guo, 2018), and prose (Zeman et al., 2013). These findings indicated that the process of appreciating Chinese calligraphy involved the perception of the visual words. Prior studies also found that the dorsal lateral prefrontal cortex (dlPFC) played a critical role in aesthetic judgments related to executive functions, especially in orienting and sustaining attention (Ferrari et al., 2015). For example, Cattaneo et al. (2010) found that the aesthetic preference for paintings could be strengthened by applying transcranial direct current stimulation (tDCS) over the dlPFC.

More importantly, prior imaging evidence has indicated that the perception of beauty elicited planning and intentions related to movements (Zhang et al., 2017, 2019). We found that the superior parietal lobule (SPL), premotor, and paracentral lobule were more significantly activated by the expert's/novice's work in conditions with AO. Observing action performed by others’ elicited activity in a network of sensorimotor brain regions collectively called the Action Observation Network (AON; Caspers et al., 2010). The core brain regions that compose the AON primarily contain the premotor and parietal cortex (see a meta‐analysis by Hardwick et al., 2018). Prior studies indicated that SPL was a crucial area for sensorimotor integration involved in action imitation and further provided a kinesthetic blueprint during AO (Hardwick et al., 2018; Krüger et al., 2014; Molenberghs et al., 2009). Similarly, the premotor cortex was primarily involved in motor planning and preparation (Cunnington et al., 2006; Schubotz & von Cramon, 2002). For example, Urgesi et al. (2004) found that the premotor cortex played a critical role in the understanding of complete body postures. Besides, we also observed that the paracentral lobule was activated in the contrast of “AO_EW > SL,” which was concerned with motor function and called the supplementary motor area (Amiez & Petrides, 2014; Zhang et al., 2017). These results suggested that the agent's hand might induce inner action imitation when the participants were appreciating calligraphy images in the condition of AO, especially observing the expert's work. In other words, AO could embody the action articulated by the creator within the participant's own motor system, which was consistent with the embodied simulation account of aesthetic experience (Freedberg & Gallese, 2007).

Notably, previous functional neuroimaging studies have found that a cortical network consisted of the cingulate gyrus and insular cortex involved the processing of emotional and rewarding processing (Di Dio et al., 2016; Boccia et al., 2016; Skov & Nadal, 2020). For example, Ishizu and Zeki (2017) proposed that the ventral ACC (vACC) was involved in the processing of emotion, especially positive emotion. Yeh et al. (2015) showed that subjective beauty and positive emotion were both related to the activation of the right ACC. Brown et al. (2011) suggested that the ACC was involved in emotional state monitoring while experiencing art. These findings have shown that the cingulate gyrus involved the processing of positive aesthetic experience. In our imaging results, we observed the cingulate gyrus activation in the contrast of “AO_EW > nonAO_EW,” which was consistent with the interpretation that the participants experienced more positive aesthetic experience when they observed the expert's work with action observation than without action observation. Significantly, we observed both regions of right ACC [6, 15, 27], left [−42, 0, 3], and right [33, 15, 6] insula were activated in the contrast of “AO_EW > nonAO_EW,” in which the regions were similar to the results of previous studies in MNI coordinates. For example, two meta‐analyses of imaging studies on aesthetic appraisal reported activation of ACC [5, 22, 19] (Kühn & Gallinat, 2012), left [−28, 0, 4] and right insula [32, 14, 2] (Brown et al., 2011). Besides, numerous previous studies have shown that insula was crucially implicated aesthetic experience, such as in everyday designed products (Yeh et al., 2015), sculptures (Di Dio et al., 2011), poems (Gao & Guo, 2018; Eugen et al., 2017), paintings (Albert et al., 2014; Cupchik et al., 2009; Di Dio et al., 2017; Ishizu & Zeki, 2013 ), and human body (Holliday et al., 2011). Therefore, the activation of the insula in our study as well suggested that it might be involved in the generation of positive aesthetic experience when the participants observed Chinese calligraphy images, especially in condition of the experts’ work with AO.

## 5 LIMITATIONS

Some limitations of the present study should be pointed out. First, we chose still images as a condition of AO instead of short films as Avanzino et al. (2015), Kirsch et al. (2016), and Mierowsky et al. (2020) did, which are more likely to provide the actual situation of AO. Therefore, we could try to use video as a stimulus to investigate the neural mechanism of the influence of the observation of artistic creation on the aesthetic experience of the observer in future studies. Second, the authors are aware that, compared with no hands for the without AO, adding the agent's hands themselves for the AO condition are more likely to activate brain areas related to viewing body parts (Peelen & Downing, 2004; Schwarzlose et al., 2005). Future studies could further control the possible confusion of the agent's hands. For example, we can add the agent's grasping the brush‐pen or flatting the hand in the control condition. We can also require the agent to correctly maintain the pen grip as for the control condition. The difference is that the agent is correctly holding some other object that is similar in shape to the brush‐pen but not related to writing, such as a wooden stick. We hope such manipulation of the control material to be effective in controlling the effects of other cues, such as stimulus size, smoothing, and even the width of calligraphic strokes. Third, previous researchers have suggested that aesthetic judgments were based on feelings of pleasure, which could be distinguished from cognitive judgments based on perception, e.g., about the brightness of objects (Ishizu & Zeki, 2013; Tsukiura & Cabeza, 2011). Given previous studies experiment design (Tsukiura & Cabeza, 2011; Zhang et al., 2016, 2017, 2020), we chose SL as the control condition in our study. However, the physical properties (e.g., contrast, spatial frequency, shape, and meaning) between calligraphy images and the SL are very different, which possibly affect the results of the contrast.

## 6 CONCLUSIONS

In sum, our previous hypothesis was confirmed by experimental evidence from behavioral and imaging. Our behavioral results indicated that appreciation of Chinese calligraphy with AO led to higher feelings of “like” than without AO. Exploring the relationship between AO and aesthetic experience thus helped us to better understand the neural substrates of aesthetic appreciation of Chinese calligraphy, especially the affective responses to the observation of the calligrapher's on‐the‐spot creation. Additionally, we found increased activation in the right SPL, the right ACC, and the bilateral insula during appreciation the expert's work with AO when compared with without AO. These regions suggested that observing creators’ creative action may evoke observers’ inner action imitation, meanwhile, further contribute to their aesthetic judgment. Moreover, our study provided preliminary evidence to support the view that not only movement characteristics (e.g., real movement and dynamic movement) implied in the content of objects but also the creative action of creators could positively affect observers’ aesthetic experience. Meanwhile, our study suggested that the aesthetic processing of Chinese calligraphy was similar to other forms of visual art, involving perceptual, cognitive, and emotional processes.

## CONFLICTS OF INTEREST

The authors declared that the research was conducted in the absence of any commercial or financial relationships that could be construed as a potential conflict of interest.

## AUTHOR CONTRIBUTIONS

Mingcheng He and Xianyou He designed the experiments and drafted the article; Wei Zhang contributed to data preprocessing and revised this manuscript critically; Jiamin Deng for data collection and making experimental materials.

### PEER REVIEW

The peer review history for this article is available at https://publons.com/publon/10.1002/brb3.2265.

## Data Availability

The data that support the findings of this study are available from the corresponding author upon reasonable request.
